# Awareness and Perspectives of Doctors on Needle Stick Injuries at Port Sudan Teaching Hospital: A Quality Improvement Project

**DOI:** 10.7759/cureus.95617

**Published:** 2025-10-28

**Authors:** Alnazeer Y Abdelbagi, Awad Abdalla Widaa Mohamed, Ahmed Shakir Ali Yousif, Asma Ahmed Osman Mohamed, Mohammed Abdalla, Shiraz Saeed Abdelrahim Saeed, Manhal Eisa Galal Eisa, Mohammed Osman Ahmed Osman, Alshyma Alfatih Mohamed Abdallah, Amany Amin Ali Hussien, Klimat Alataya Ibrahim Adam, Manasik Abdaldeen Mohamed Ahmed, Thoiba Mohammed Hamdnaallah Mohammed, Haifa Yousif Mohamed Elhaj, Mohamed Abdalla Elawad Wedatalla, Mohey Aldien Ahmed Elamin Elnour, Sara Y Mukhtar, Eman Esam Hassan Ali, Hadeel Omer Elhag Ahmed, Samah Elhussein Mohamed Ahmed

**Affiliations:** 1 Department of Surgery, Port Sudan Teaching Hospital, Port Sudan, SDN; 2 Department of General Surgery, Port Sudan Teaching Hospital, Port Sudan, SDN; 3 Department of Urology, Port Sudan Teaching Hospital, Port Sudan, SDN; 4 Department of Medicine, Port Sudan Teaching Hospital, Port Sudan, SDN; 5 Department of Internal Medicine, Port Sudan Teaching Hospital, Port Sudan, SDN; 6 Department of Trauma and Orthopedics, Port Sudan Teaching Hospital, Port Sudan, SDN

**Keywords:** bloodborne pathogens, educational interventions, healthcare workers, needlestick injuries, occupational health, postexposure prophylaxis, quality improvement, reporting culture, sharps safety, training

## Abstract

Background: Needlestick injuries (NSIs) pose a threat to healthcare workers (HCWs) by exposing them to bloodborne pathogens. Irrespective of safety devices and policies, knowledge, practice, and reporting gaps still exist. This study aimed to audit the knowledge, attitudes, and practices of HCWs regarding NSIs in a tertiary hospital and to determine the effects of educational and system-level interventions.

Methods: A two-cycle quality-improvement audit was held at Port Sudan Teaching Hospital. Cycle 1 defined compliance at the baseline level through review of documents, staff surveys, and observation. Interventions were education sessions, practical workshops, quick-reference materials, better access to safety-engineered devices, and promotion of a nonpunitive reporting culture. Repeat measurements were conducted in Cycle 2 using the same standards: safety device use, no recapping, sharps container practices, immediate first aid, reporting, and postexposure prophylaxis (PEP) procedures. A total of 97 HCWs participated (Cycle 1: n = 53; Cycle 2: n = 44).

Results: The interventions produced significant improvements. Recognition of hepatitis B virus as a bloodborne risk increased from 33/53 (62.3%) to 39/44 (88.6%) (p = 0.003). Correct initiation of PEP within 72 hours rose from 18/53 (34.0%) to 34/44 (77.3%) (p < 0.001). Safety behaviors improved markedly: use of safety-engineered devices increased from 17/53 (32.1%) to 35/44 (79.5%) (p < 0.001); needle recapping decreased from 21/53 (39.6%) to 2/44 (4.5%) (p < 0.001); and correct use of sharps boxes (two-thirds full) increased from 15/53 (28.3%) to 34/44 (77.3%) (p < 0.001). Training uptake rose significantly: formal training increased from 6/53 (11.3%) to 21/44 (47.7%) (p < 0.001), and refresher training from 2/53 (3.8%) to 12/44 (27.3%) (p = 0.001). Awareness of a formal reporting system improved from 8/53 (15.1%) to 21/44 (47.7%) (p < 0.001). However, awareness of the hepatitis C virus and human immunodeficiency virus (HIV) as risk factors and some unsafe first-aid practices persisted as areas requiring further strengthening.

Conclusions: An education-based approach, reinforced by practical training, improved device availability, and a favorable reporting culture, led to significant improvements in NSI knowledge and safe practice. Continued refresher education and stronger communication about HCV/HIV risks are required to maintain and extend gains. Limitations include the single-center design, reliance on self-reports, and potential underreporting.

## Introduction

Needlestick injuries (NSIs) are among the most significant occupational hazards faced by healthcare workers (HCWs) worldwide, particularly those involved in performing invasive procedures. These injuries involve accidental skin punctures caused by needles or other sharp medical instruments contaminated with blood or body fluids, posing a risk of transmission of serious bloodborne pathogens such as hepatitis B virus (HBV), hepatitis C virus (HCV), and human immunodeficiency virus (HIV) [1].

Globally, it is estimated that over two million NSIs occur annually among HCW, resulting in approximately 66,000 HBV, 16,000 HCV, and 1,000 HIV infections each year [2]. According to the World Health Organization (WHO), about 40% of HBV and HCV infections and 2.5% of HIV infections among healthcare professionals are attributable to occupational exposures [3]. Despite advances in safety-engineered devices and infection control measures, NSIs remain underreported, particularly in low- and middle-income countries.

In Sudan and other low-resource settings, limited surveillance systems and inconsistent reporting practices contribute to a lack of accurate national data; however, local studies indicate that between 40% and 70% of HCWs experience at least one NSI during their career, with underreporting rates exceeding 60% [4,5]. These high rates reflect gaps in awareness, workload pressures, and inadequate training.

Knowledge of NSI risks, adherence to safety protocols, and timely reporting of incidents are critical to prevention and postexposure management [6]. Although precautionary measures and institutional policies are widely promoted, studies continue to reveal deficiencies in knowledge and variable attitudes toward NSI prevention and reporting among HCWs [7]. Such gaps increase vulnerability to occupational infections and compromise postexposure management practices [8].

Clinical audits assessing awareness, attitudes, and practices among HCWs are essential to identify behavioral, educational, and systemic barriers. The present audit aimed to evaluate the knowledge, attitudes, and practices of HCWs regarding NSIs in a tertiary hospital, with the ultimate goal of enhancing compliance with safe practices and promoting occupational safety [9]. National and institutional infection control frameworks provide structured guidance for managing NSIs and postexposure prophylaxis (PEP) [10].

## Materials and methods

Study design and setting

The purpose of this quality improvement clinical audit at Port Sudan Teaching Hospital was to assess the occurrence, reported and unreported cases, and management of NSIs in HCWs. The mixed retrospective and prospective observational design was used to measure routine practice in two audit cycles to evaluate the effectiveness of the interventions that followed afterward.

Study population

The study population comprised all HCWs at Port Sudan Teaching Hospital who were at risk of occupational exposure to blood-borne infections during clinical procedures. This included doctors, nurses, laboratory technicians, and ancillary staff working in surgical, medical, emergency, and laboratory departments.

At the time of the audit, the hospital employed approximately 120 HCWs in these clinical areas. A total of 97 HCWs participated in the study, yielding a response rate of 80.8%. Of these, 53 participants took part in Cycle I (baseline) and 44 participants in Cycle II (postintervention). Data collection involved review of incident reports, administration of structured staff surveys, and direct observation of sharps handling practices.

The structured questionnaire used for data collection (see Appendix 1) was developed by the authors based on published literature and international occupational safety guidelines. It comprised sections assessing knowledge, attitudes, reporting behavior, and preventive practices related to NSIs.

The questionnaire was reviewed by a specialist in orthopedic surgery for content validity, clarity, and clinical relevance to surgical practice. Minor modifications were made based on the specialist’s feedback before administration. The tool was not formally validated through psychometric testing, and this has been acknowledged in the study limitations.

Audit standards and criteria

The international and institutional occupational health and sharps safety standards that were used to develop the audit criteria were adapted from the guidelines of the National Institute for Health and Care Excellence (NICE) [9], the WHO [10], and the Port Sudan Teaching Hospital infection control policy [11].

The parameters assessed included the utilization of safety-engineered equipment, compliance with the “no recapping” policy, the presence and correct utilization of sharps containers, and staff training that had taken place within the previous year. Immediate exposure management was also considered, including the provision of appropriate first aid at the site, reporting the incident within one hour, and completing a risk assessment.

Record keeping was reviewed to confirm documentation of source patient testing and baseline testing of exposed workers within 24 hours. Practices related to PEP were also examined, including HIV PEP initiation, HBV prophylaxis, and adherence to HCV follow-up protocols [12,13]. The adequacy of follow-up, compliance with regulatory reporting requirements, and performance of root cause analyses were additionally audited [14].

First cycle baseline audit and problem identification (May 17, 2025, to June 30, 2025)

In the initial audit cycle, compliance with the above standards was assessed through examination of available documentation and responses of staff. The results of the baseline showed the low adoption rates of safety apparatus, the common nonobservation of disposal policies, and uneven knowledge of postexposure management procedures. Junior doctors lacked confidence in the management of NSIs, and underreporting of NSIs was a common occurrence. According to informal interviews and observations, inadequate structured training, the lack of the availability of safety-engineered equipment, and the low level of reporting culture were the key obstacles.

Intervention: education and system strengthening (July 01, 2025, to July 15, 2025)

According to the root cause analysis, a number of interventions were undertaken. Training was performed to refresh the knowledge on prevention, reporting, and management instructions. Raising awareness about the safe handling of sharps and postexposure was done through practical workshops. Reminders were in the form of quick-reference guides and posters that were located in clinical areas. There was an improvement in access to safety-engineered sharp devices and properly maintained sharps bins. In addition, the introduction of a new reporting system (see Appendix 2) helped promote a culture of nonpunitive reporting by streamlining the reporting process and reinforcing its importance through targeted training programs.

Second cycle: postintervention audit and outcome assessment (July 16, 2025, to September 01, 2025)

The intervention's effect was assessed in the second audit cycle, which was also conducted in the same manner. Significant changes were seen. The use of safety-engineered devices rose significantly, education in the use of sharps boxes rose considerably, and attendance in training and refresher training courses rose significantly. Healthcare employees also reported being more confident in and ready to handle NSIs, and knowledge about the existence of the formal reporting system increased, yet there was still the issue of underreporting.

Data analysis

Data were entered and analyzed using the Statistical Package for the Social Sciences version 24.0 (IBM Corp., Armonk, NY). Descriptive statistics were used to summarize categorical variables as frequencies and percentages. The chi-square test (χ²) was applied to compare proportions between the two audit cycles (Cycles I and II) to assess changes in compliance with audit standards before and after interventions. A p value of <0.05 was considered statistically significant. Results were presented in tabular and graphical formats to illustrate the degree of improvement across the evaluated parameters.

Ethical considerations

This quality improvement audit involved HCWs at Port Sudan Teaching Hospital and did not include patients or the collection of human tissue. Participation was voluntary, and informed consent was obtained from all respondents prior to data collection. The audit was reviewed and approved by the Port Sudan Teaching Hospital Ethics Committee, which granted an approval/waiver in accordance with institutional and national guidelines on clinical audits and quality improvement projects. All data were anonymized to protect confidentiality.

## Results

A total of 97 HCWs participated in both audit cycles, with 53 in Cycle 1 and 44 in Cycle 2. Compliance with knowledge, immediate first aid, PEP, needlestick safety practices, and training measures showed marked variation between the two cycles.

Knowledge of bloodborne pathogens improved for HBV, increasing from 33/53 (62.3%) in Cycle 1 to 39/44 (88.6%) in Cycle 2. This was a statistically significant finding (χ² = -2.96, p = 0.003, 95% CI = -42.4% to -10.3%). By contrast, awareness of HCV declined from 5/53 (9.4%) in Cycle 1 to 1/44 (2.3%) in Cycle 2 (p = 0.145). Similarly, identification of HIV as a risk pathogen decreased from 12/53 (22.6%) in Cycle 1 to 4/44 (9.1%) in Cycle 2 (p = 0.073). The proportion of respondents who admitted not knowing the pathogen risk dropped from 3/53 (5.7%) in Cycle 1 to none in Cycle 2.

With regard to immediate first-aid measures, washing the site with soap and water improved from 41/53 (77.4%) in Cycle 1 to 40/44 (90.9%) in Cycle 2, although this difference did not reach statistical significance (p = 0.073). Unsafe practices, including immediate bandaging, decreased from 7/53 (13.2%) in Cycle 1 to 1/44 (2.3%) in Cycle 2 (p = 0.051). Wound sucking declined slightly from 4/53 (7.5%) in Cycle 1 to 2/44 (4.5%) in Cycle 2 (p = 0.541). Doing nothing was reported by 1/44 (2.3%) in Cycle 2 compared with none in Cycle 1.

In terms of PEP knowledge, awareness of the correct definition of PEP rose from 37/53 (69.8%) in Cycle 1 to 36/44 (81.8%) in Cycle 2, though this was not statistically significant (p = 0.172). A substantial and statistically significant improvement was observed in recognition of correct initiation within 72 hours, which increased from 18/53 (34.0%) in Cycle 1 to 34/44 (77.3%) in Cycle 2 (χ² = -4.26, p < 0.001, 95% CI = -61.1% to -25.5%).

Significant progress was also reported in relation to safety practices. The use of safety-engineered devices increased from 17/53 (32.1%) in Cycle 1 to 35/44 (79.5%) in Cycle 2 (p < 0.001). Recapping of needles, considered an unsafe practice, declined sharply from 21/53 (39.6%) in Cycle 1 to only 2/44 (4.5%) in Cycle 2 (p < 0.001). Compliance with correct sharps disposal in a two-thirds full safety box improved from 15/53 (28.3%) in Cycle 1 to 34/44 (77.3%) in Cycle 2 (p < 0.001). A composite measure reflecting no recapping with proper disposal rose from none of the 53 participants in Cycle 1 (0.0%) to 30/44 (68.2%) in Cycle 2 (p < 0.001). By contrast, recapping followed by disposal fell from 33/53 (62.3%) in Cycle 1 to 13/44 (29.6%) in Cycle 2 (p = 0.001).

Training and awareness also showed substantial progress. The number of participants who received formal training on NSI prevention increased from 6/53 (11.3%) in Cycle 1 to 21/44 (47.7%) in Cycle 2 (p < 0.001). Regular refresher training rose significantly from 2/53 (3.8%) in Cycle 1 to 12/44 (27.3%) in Cycle 2 (p = 0.001). At the same time, the proportion who never had refresher training declined from 41/53 (77.4%) in Cycle 1 to 23/44 (52.3%) in Cycle 2 (p = 0.009). The perceived adequacy of training rose sharply, with only 3/53 (5.7%) in Cycle 1 reporting that they felt adequately trained compared with 27/44 (61.4%) in Cycle 2 (p < 0.001). Awareness of a formal reporting system similarly improved from 8/53 (15.1%) in Cycle 1 to 21/44 (47.7%) in Cycle 2 (p < 0.001).

Suggested improvements identified by participants also demonstrated notable changes. Requests for more training and education rose from 46/53 (86.8%) in Cycle 1 to 44/44 (100.0%) in Cycle 2 (p = 0.012). Better access to safety devices was advocated by 30/53 (56.6%) in Cycle 1 and by 38/44 (86.4%) in Cycle 2 (p = 0.001). Demand for regular monitoring and feedback increased from 18/53 (34.0%) in Cycle 1 to 30/44 (68.2%) in Cycle 2 (p = 0.001). The proportion supporting a more supportive reporting culture rose from 17/53 (32.1%) in Cycle 1 to 26/44 (59.1%) in Cycle 2 (p = 0.008). Advocacy for university-based sessions remained low, with only 1/53 (1.9%) in Cycle 1 and 2/44 (4.5%) in Cycle 2 (p = 0.451).

In summary, the audit cycles demonstrated statistically significant improvements in HBV recognition, correct and timely PEP initiation, safety-engineered device use, elimination of needle recapping, correct sharps disposal, training uptake, perceived adequacy of training, and awareness of reporting systems. At the same time, unsafe practices such as needle recapping and immediate bandaging declined markedly. Despite these gains, knowledge regarding HIV and HCV as pathogens declined between cycles, and a minority of respondents continued to report unsafe immediate first-aid measures, highlighting the need for targeted reinforcement in these specific areas (Table 1).

**Table 1:** Compliance with needlestick injury knowledge, practices, and reporting before and after interventions at Port Sudan Teaching Hospital CI: confidence interval; HBV: hepatitis B virus; HCV: hepatitis C virus; HIV: human immunodeficiency virus; NSI: needlestick injury; PEP: postexposure prophylaxis

Parameter	Compliance (cycle 1)	Compliance (cycle 2)	% Improvement	χ²	p value	95% CI
HBV identified as pathogen	62.3% (33/53)	88.6% (39/44)	26.4	-2.96	0.003	-42.4 to -10.3
HCV identified	9.4% (5/53)	2.3% (1/44)	-7.2	1.46	0.145	-1.9 to 16.2
HIV identified	22.6% (12/53)	9.1% (4/44)	-13.6	1.79	0.073	-0.6 to 27.7
I don't know (pathogen)	5.7% (3/53)	0.0% (0/44)	-5.7	1.60	0.109	-0.6 to 11.9
Correct first step (wash with soap and water)	77.4% (41/53)	90.9% (40/44)	13.5	-1.79	0.073	-27.7 to 0.6
Bandage immediately	13.2% (7/53)	2.3% (1/44)	-10.9	1.95	0.051	0.8 to 21.1
Suck wound	7.5% (4/53)	4.5% (2/44)	-3.0	0.61	0.541	-6.4 to 12.4
Do nothing	0.0% (0/53)	2.3% (1/44)	2.3	-1.10	0.270	-6.7 to 2.1
PEP correctly defined	69.8% (37/53)	81.8% (36/44)	12.0	-1.36	0.172	-28.8 to 4.8
Correct PEP initiation within 72 hours	34.0% (18/53)	77.3% (34/44)	43.3	-4.26	0.000	-61.1 to -25.5
Safety devices used	32.1% (17/53)	79.5% (35/44)	47.5	-4.67	0.000	-64.8 to -30.2
Recapping needles	39.6% (21/53)	4.5% (2/44)	-35.1	4.04	0.000	20.5 to 49.6
Correct sharps box use (2/3 full)	28.3% (15/53)	77.3% (34/44)	49.0	-4.80	0.000	-66.3 to -31.6
No recap and sharps disposal	0.0% (0/53)	68.2% (30/44)	68.2	-7.23	0.000	-81.9 to -54.4
Recap and sharps disposal	62.3% (33/53)	29.6% (13/44)	-32.7	3.21	0.001	14.0 to 51.5
Trash bin disposal	3.8% (2/53)	0.0% (0/44)	-3.8	1.30	0.193	-1.4 to 8.9
Received formal NSI training	11.3% (6/53)	47.7% (21/44)	36.4	-3.98	0.000	-53.5 to -19.4
Regular refresher training	3.8% (2/53)	27.3% (12/44)	23.5	-3.28	0.001	-37.6 to -9.4
Occasional refresher training	18.9% (10/53)	20.4% (9/44)	1.6	-0.20	0.845	-17.5 to 14.3
Never refresher training	77.4% (41/53)	52.3% (23/44)	-25.1	2.60	0.009	6.5 to 43.7
Feel adequately trained	5.7% (3/53)	61.4% (27/44)	55.7	-5.91	0.000	-71.4 to -40.0
Aware of formal reporting system	15.1% (8/53)	47.7% (21/44)	32.6	-3.50	0.000	-50.3 to -15.0
More training and education (suggestion)	86.8% (46/53)	100.0% (44/44)	13.2	-2.50	0.012	-22.3 to -4.1
Better safety device access (suggestion)	56.6% (30/53)	86.4% (38/44)	29.8	-3.19	0.001	-46.5 to -13.0
Regular monitoring and feedback (suggestion)	34.0% (18/53)	68.2% (30/44)	34.2	-3.36	0.001	-53.0 to -15.5
Supportive reporting culture (suggestion)	32.1% (17/53)	59.1% (26/44)	27.0	-2.67	0.008	-46.2 to -7.8
University-based sessions (suggestion)	1.9% (1/53)	4.5% (2/44)	2.6	-0.75	0.451	-9.8 to 4.5

## Discussion

This audit shows that there were substantial changes in the knowledge, attitudes, and practices of HCWs in response to NSIs at Port Sudan Teaching Hospital following structured educational and system-level interventions. The findings are in line with global findings that training, system reinforcement, and the provision of safety devices translate into quantifiable improvements in occupational safety practices [1-3].

HBV was characterized as a bloodborne pathogen, and awareness of this pathogen increased significantly from 62.3% to 88.6% in the first and second cycles, respectively, which was statistically significant (p = 0.003). This is in line with past research, where HBV is most commonly detected through mass vaccination and institutional focus [1,3]. Nonetheless, HCV knowledge decreased from 9.4% to 2.3% (p = 0.145), and HIV awareness declined from 22.6% to 9.1% (p = 0.073). Although these changes were not statistically significant, several contextual factors may explain this pattern. Cycle II participants were drawn from the same population of HCWs but were not identical individuals, as some staff had rotated or transferred to other hospitals during the audit period. This staff turnover may have introduced new participants with lower baseline knowledge. Additionally, the training sessions placed greater emphasis on HBV prevention and vaccination, reflecting institutional priorities, resulting in less focus on HIV and HCV content. Limited local resources, low perceived risk, and restricted access to updated HIV/HCV post-exposure guidelines may have also contributed to these persistent knowledge gaps. These findings are consistent with other studies showing that HCWs often underestimate the occupational risks associated with HCV and HIV despite their clinical importance [4,5]. This implies that there should be an equal and consistent form of education reinforcement to ensure that all bloodborne pathogens are well known.

There was an improvement in first-aid practices, with the percentage of washing with soap and water increasing to 90.9% (p = 0.073) vs. 77.4%, and inappropriate practices such as immediate bandaging (13.2%-2.3%) and sucking of the wound (7.5%-4.5%) decreasing (p = 0.051). All the changes were not statistically significant; however, the general pattern indicates a positive effect of the intervention. Such findings can be compared to audits in which standardized displays and active workshops minimized unsafe reactions following NSI [2,6].

There was also an improvement in PEP knowledge. There was an increase in awareness of the definition of PEP (69.8%-81.8%; p = 0.172) and recognition of the initiation within 72 hours (34.0%-77.3%, highly significant change, p < 0.001). This gain is clinically significant, as initiating PEP as soon as possible is correlated with a lower risk of seroconversion following occupational exposure [7,8].

The greatest improvement was recorded in Sharps safety practices. The proportion of safety-engineered devices grew by 32.1%-79.5% (p < 0.001), whereas the proportion of needle recapping dropped sharply by 39.6%-4.5% (p < 0.001). The proportion of correct sharps box use increased (28.3%-77.3%) (p < 0.001), and composite measures of safe practice (no recap with proper disposal) increased (0%-68.2%) (p < 0.001). Unsafe measures, such as dumping in garbage bins, were done away with, although there were some instances of failure. These results underscore the effectiveness of both training and better access to devices, aligning with the literature that demonstrates a combination of education and resources is most effective in decreasing unsafe disposal [1,3,4].

The adoption rates of training also increased, with formal training becoming 47.7% as opposed to 11.3% (p < 0.001) and refresher training becoming 27.3% as opposed to 3.8% (p = 0.001). The percentage of those who never received a refresher training decreased to 52.3% (p = 0.009). The perception of sufficient training had significantly increased from 5.7% to 61.4% (p < 0.001), indicating more confidence in the staff. Knowledge of formal reporting systems increased (15.1%-47.7%) (p < 0.001), yet underreporting remained an issue. Feedback on staff also identified that cultural and systems barriers, including fear of blame or no feedback, may continue to deter reporting. This is in accordance with other contexts, where nonpunitive environments and affirmative reporting cultures have proved paramount in conquering these obstacles [3,6,8].

Incident reporting also demonstrated notable improvement following the intervention. Awareness and correct understanding of the reporting process increased markedly between audit cycles, reflecting greater willingness to report occupational exposures. However, this study focused on knowledge and self-reported practices, rather than the formal count of submitted incident reports. Therefore, while improved awareness suggests a positive behavioral trend, underreporting may still exist in actual hospital records. Ongoing monitoring and reinforcement of a nonpunitive reporting culture remain essential.

A key strength of this audit is its two-cycle design, which allowed direct comparison of pre- and postintervention outcomes using international standards (WHO and NICE). However, several limitations should be acknowledged. The study was conducted at a single center with a small sample size (n = 97), which may limit generalizability. Data were self-reported, creating potential for recall bias and social desirability bias, as participants may have overreported compliance with safety practices or underreported unsafe behaviors. In addition, staff shifting and turnover occurred during the audit period, as some HCWs were transferred or rotated to other hospitals. Consequently, although the 44 participants in Cycle II were drawn from the same population of HCWs as those in Cycle I, they were not necessarily the same individuals, which may have introduced variability in comparative results. The questionnaire, while reviewed by a specialist in orthopedic surgery for content validity, was not formally validated or pilot-tested. Furthermore, selection bias and uncontrolled confounding factors cannot be excluded. Despite these limitations, the statistically significant improvements observed strongly support the effectiveness of the educational and system-level interventions.

Practically, these results imply that sustained learning, enhanced availability of safety-designed equipment, and a culture of reporting are essential in maintaining progress in NSI prevention and control. Although national and institutional policies on infection control are necessary guidelines, their effectiveness is seen in how well they are implemented at the local level [7]. The continuity of the lack of awareness on HIV and HCV and reporting practices indicates that specific reinforcement measures and long-term follow-up should be implemented to ensure that the positive changes will be sustained and prolonged.

## Conclusions

This audit shows that the systematic education program, with the support of practical workshops, increased supply of safety equipment, and the development of a favorable culture of reporting, can significantly increase the knowledge, attitudes, and practices of HCWs on the subject of NSIs. The results show that there was a substantive increase in HBV awareness, safe sharps disposal, 72-hour PEP uptake, and staff confidence due to training. Despite this, gaps in awareness about risks of HIV and HCV, and barriers to reporting on a system level show the necessity of constant reinforcement, periodic refresher education, and system-level support. Further efforts must aim to entrench these measures as standard practice, and therefore, long-term sustainability will be realized, and local occupational safety standards will be in tandem with international standards.

## Figures and Tables

**Figure 1:**
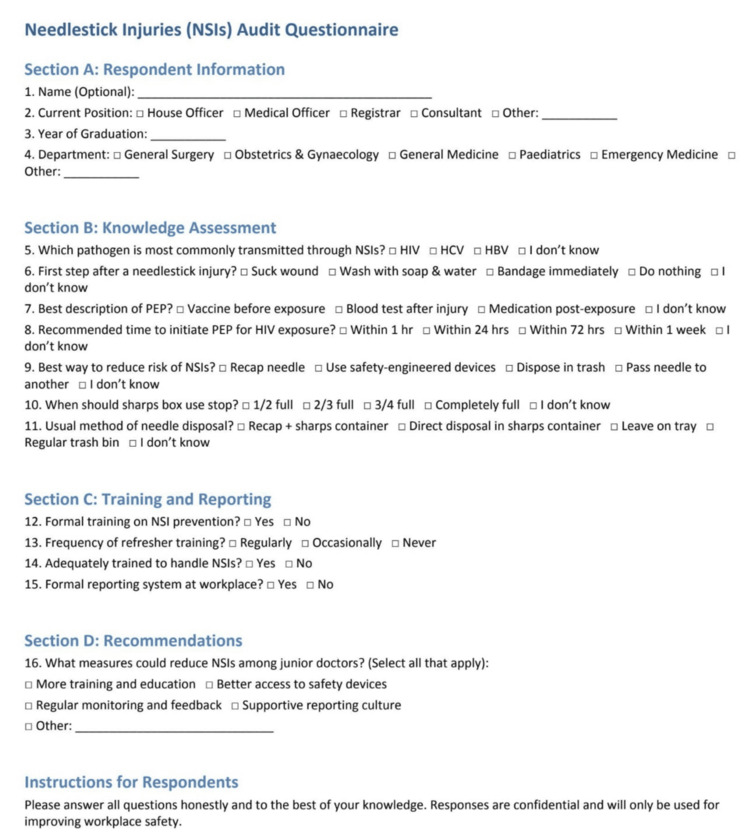
Needlestick injuries audit questionnaire

**Figure 2:**
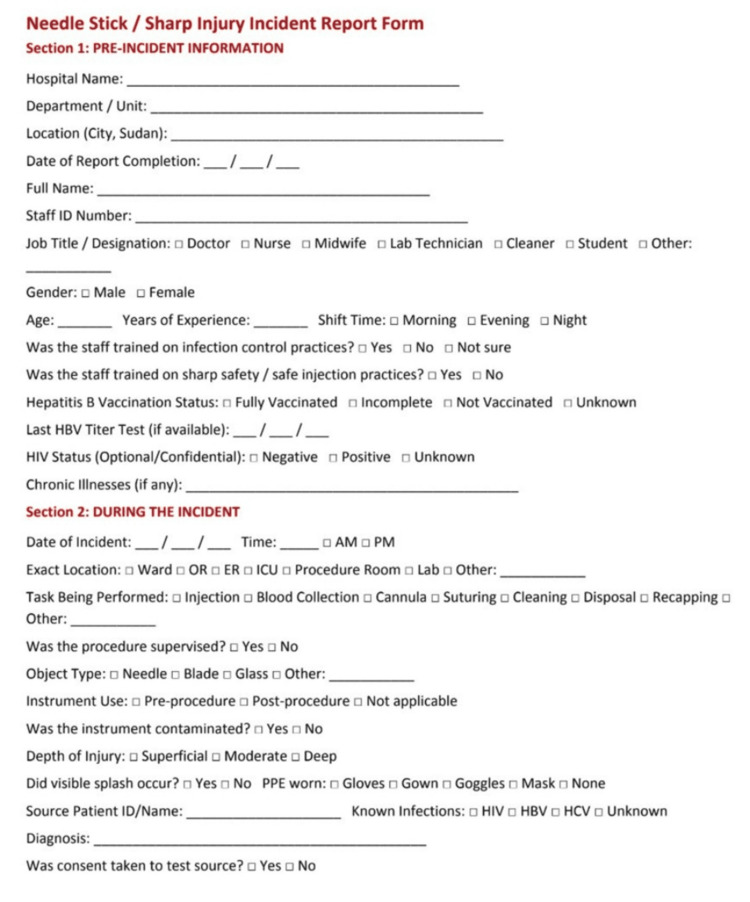
The front page of the new reporting form

**Figure 3:**
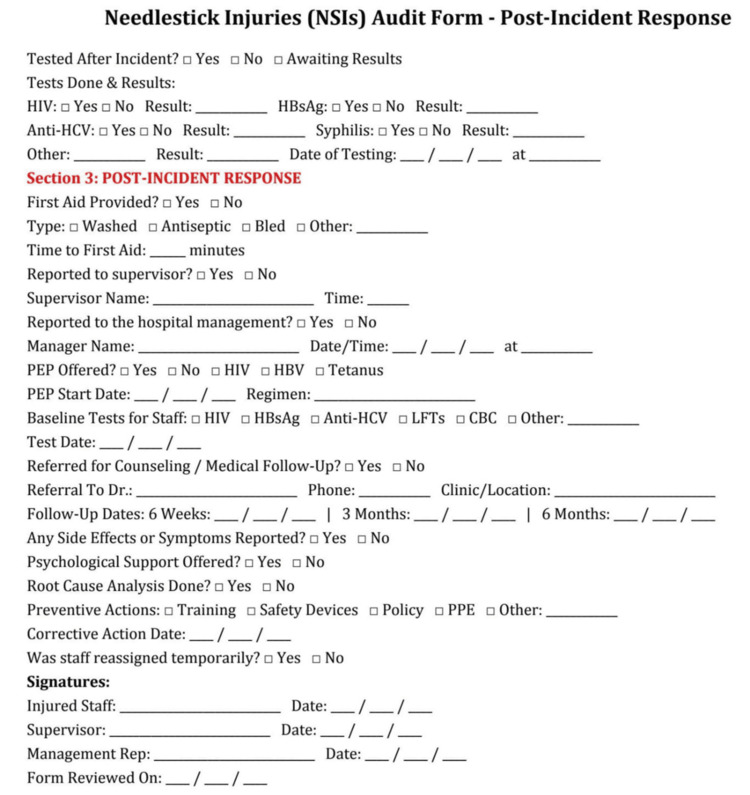
The back page of the new reporting form

## Appendices

Appendix 1

Appendix 2
